# Absolute Configuration and Chemical Topology

**DOI:** 10.6028/jres.067A.058

**Published:** 1963-12-01

**Authors:** Stephen J. Tauber

## Abstract

The stereochemistry of catenanes, knotted molecules, and Borromean rings is discussed. An augmentation of the Cahn-Ingold-Prelog convention for designating absolute configuration is proposed. A convention is proposed for designating the absolute configuration of knotted molecules. A suggestion is made concerning the citing of the absolute configuration of molecularly dissymmetric diastereomers.

Proposals of rules for designating absolute configuration have been put forth by Cahn, Ingold, and Prelog^1^ and by Terentiev and Potapov.^2^ The rules of the latter workers are deliberately linked to nomenclature. Those of the former workers rely entirely on structure and have found wide acceptance among organic chemists. In their closely reasoned paper Cahn, Ingold, and Prelog properly claim to have covered by their rules all known types of dissymmetry deriving from tetracovalent and tricovalent atoms.

Work aiming toward the establishment of a major system for handling chemical information requires the ability precisely to designate chemical structures without recourse to structural formulas. A number of notation systems have been devised for representing chemical structures by linear arrays of symbols,^3^ although none of the systems has yet been perfected even for organic compounds.^4^ Since an adequate information handling system must accommodate not only all known structural types but also those structural types which have to date been merely speculated on, an examination of more esoteric stereochemistry was undertaken. I now wish to propose an augmentation of the Cahn-Ingold-Prelog (C-I-P) rules and the addition of a new (specialized) set of rules to cover a wider range of molecularly dissymmetric structural types.

The molecularly dissymmetric structures for which further rules are necessary are of two types: (1) substituted catenanes,^5^ which are stereochemically related to substituted allenes and spiro compounds, and (2) knots,^6^, ^7^ compounds whose dissymmetry is due entirely to topology, independent of substitution. ^6, 7^ The C-I-P convention is inapplicable to knots and before being applied to catenanes requires a further convention for defining the “near groups”.

## Catenanes

**Figure 1:**
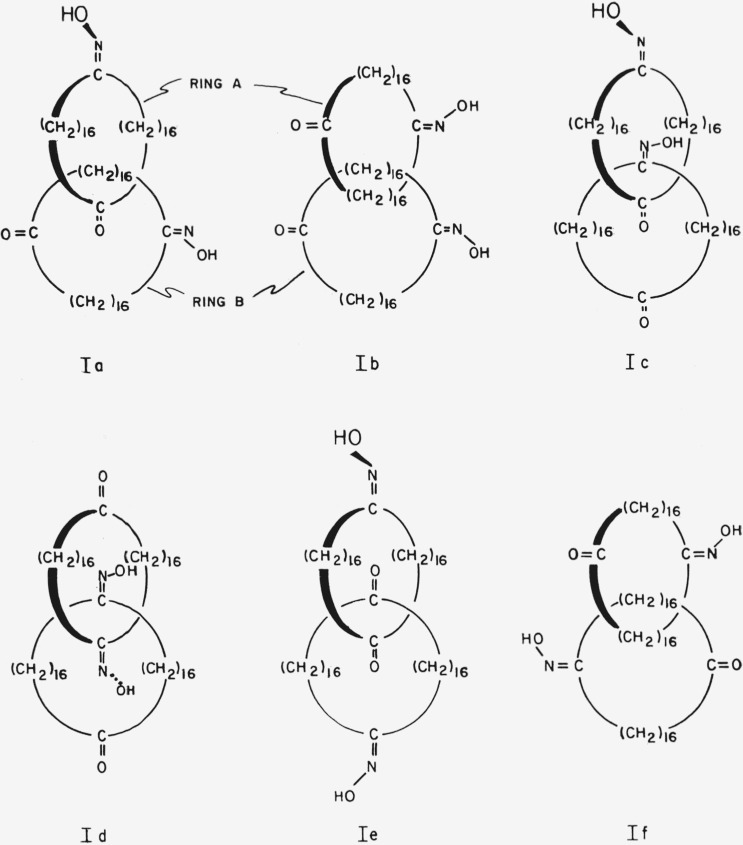
[34.34]-Catenan-1,18,1′,18′-tetraone 1,1′-dioxime in various conformations differing in the angles of rotation about the axes of the individual rings.

**Figure 2:**
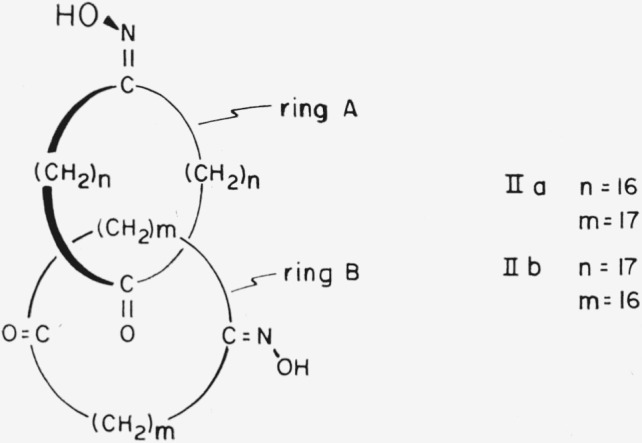
Two conformations of [36.34]-catenan-1,19,1′,18′-tetraone 1,1′-dioxime.

**Figure 3:**
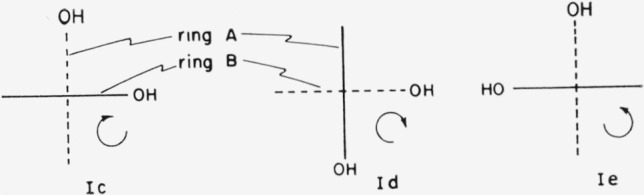
En views of [34.34]-catenan-1,18,1′,18′-tetraone 1,1′-dioxime in the conformations preferred by the Cahn-Ingold-Prelog convention. The curved arrows indicate the directions defined by the conversion rule.

Because of free rotation of each ring of a catenane about its own axis, compounds such as [34.34]-catenan-1,18,1′,18′-tetraone 1,1′-dioxime, I, of which two enantiomeric configurations are possible, could exist in any number of conformations related to the angle of rotation about the axes of the rings. Some of the conformations of one of the enantiomers are shown in figure 1. If the two rings are not identical, as in [36.34]-catenan-1,19,1′,18′-tetraone 1,1′-dioxime, II, then some of the conformations of figure 1 correspond to two distinct conformations; e.g., conformation la corresponds to conformations IIa and IIb (fig. 2). The choice of an “axis of asymmetry” under section 5.1 of Cahn et al.^1^ (such that the dissymmetry element contain the maximum number of atoms, with the widest possible distribution, and most preferred by the sequence rule) would require basing the designation of the configuration of [34.34]-catenan-1,18,1′,18′-tetraone 1,1′-dioxime on conformation Ic, Id, or Ie. Application to these three conformations of the C-I-P sequence rules does *not* lead to assignment of the same absolute configuration (cf. fig. 3).

**Figure 4:**
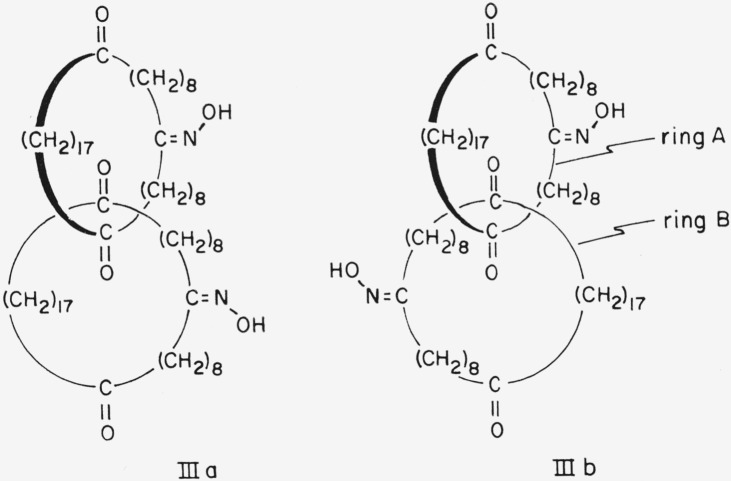
[36.36]-Catenan-1,10,19,1′,10′,19′-hexaone 10,10′-dioxime in the conformations which place the preferred atoms^1^ on a potential axis of dissymmetry, here occupied by the four carbonyl groups.

**Figure 5:**
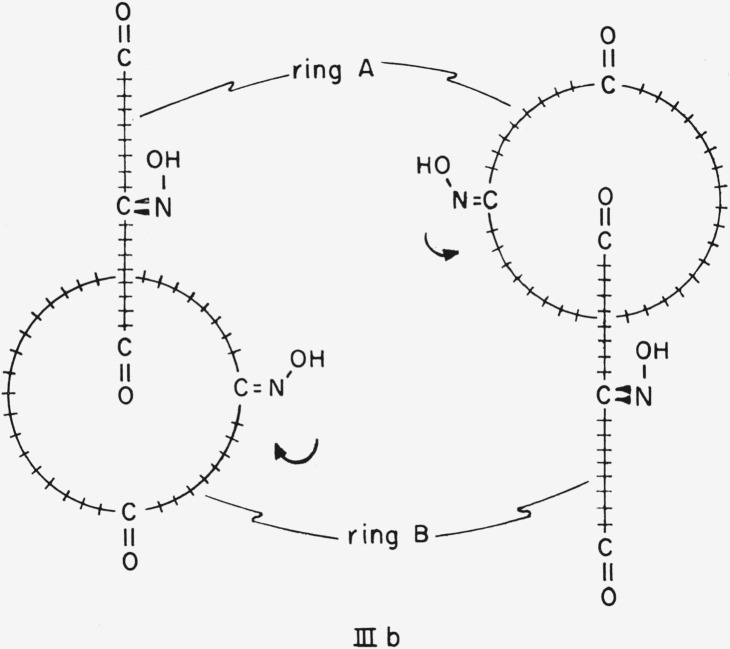
Two views of a single conformation of [36.36]-catenan-1,10,19,1′,10′,19′-hexaone 10,10′-dioxime. The curved arrows indicate the directions defined by the conversion rule.^1^

For a compound such as [36.36]-catenan-1,10,-19,1′,10′,19′-hexaone 10,10′-dioxime, III, an attempt to choose an axis of dissymmetry under section 5.1 of Cahn et al. would be thwarted because the carbonyl oxygen atoms would have to lie on the axis, and the molecule would then have a plane of dissymmetry instead of an axis of dissymmetry (cf. fig. 4). In this case it would be necessary to choose not only the appropriate side of the plane of dissymmetry from which to view the molecule but, because the three atoms specified by section 5.3 of Cahn et al.^1^ (the oxime C, N, and O—cf., below) lie in a plane perpendicular to the plane of dissymmetry, also the appropriate quadrant on that side. This ought to be the quadrant *nearer* the atom *in* the plane of dissymmetry most senior by the sequence rule. However even for a specific conformation, e.g., IIIb, the designation of absolute configuration would differ according to whether the plane of dissymmetry be chosen to contain ring A or ring B (cf. fig. 5). Note that in [36.36]-catenan-1,10,19,1′,10′,19′-hexaone 10,10′-dioxime the methylene carbon atoms, and the corresponding hydrogen atoms, are pairwise identical (positions 9 and 11,8 and 12, 2′ and 18′, etc.) under the sequence rule. It is therefore necessary to base the designation of configuration on atoms not bound directly to the plane of dissymmetry. A similar situation obtains when the considerations of section 5.3 (planar asymmetry) of Cahn et al. are applied to a compound having a polysulfide bridge instead of a polymethylene ether bridge lying in the plane of dissymmetry. Thus the atoms determining the configuration of 2,3,4,5,6,7,8,9,10,11-decathiabicyclo-[10.2.2]hexadeca-12,14,15-triene-13-carboxylic acid,^8^ IV, are the 13, 14 and carboxyl carbon atoms.

**Figure:**
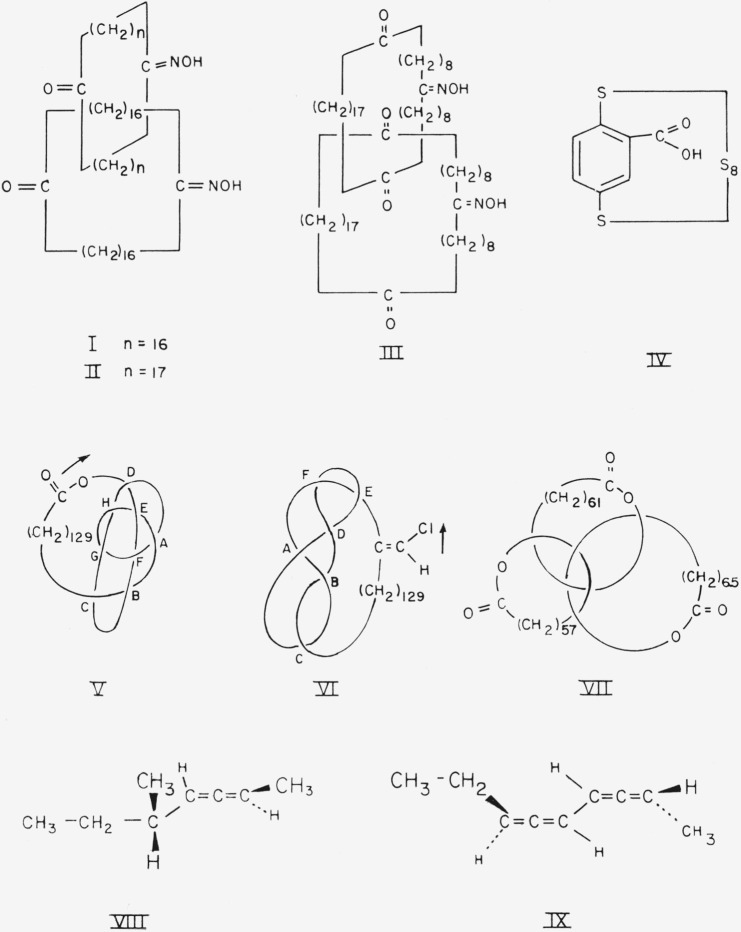


I therefore propose the following rule to govern the choice of the element of dissymmetry:

### Dissymmetry Element Rule

Select an element of dissymmetry by applying in turn the following hierarchy of criteria:

1. The element of dissymmetry be of minimum dimensionality;
2. It contain a maximum number of atoms;
3. The atoms have maximum distribution;
4. The element of dissymmetry contain the atom or set of atoms most senior by the sequence rule; and
5. The atoms on the element of dissymmetry most senior by the sequence rule have the maximum separation.

The philosophy of first difference of the C-I-P convention is maintained; the substantive changes proposed appear in italics.

**Figure 6:**
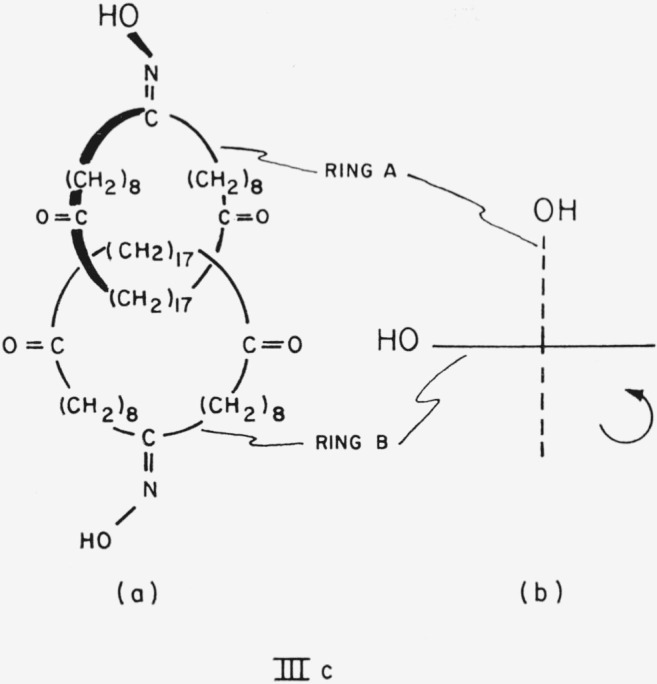
[36.36]-Catenan-1,10,19,1′,10′,19′-hexaone 10,10′-dioxime in the preferred conformation according to the presently proposed convention: (a) perspective and (b) end view. This conformation has an axis of dissymmetry.

Application of subrule (5) resolves the ambiguity with respect to compound I, causing conformation Ie to be the proper one from which to determine the absolute configuration and thus causing the enantiomer represented to be the (*S*)-form. Application of subrules (1) and (5) to compound III requires conformation IIIc (fig. 6) to be the basis for determination of the absolute configuration, and the enantiomer shown of compound III is the (*S*)-form.

## Knots

It was pointed out at least as early as 1953, by Ambs,^6^ that a simple cyclic compound could exist as an enantiomeric pair if it were in the form of a trefoil knot. The same observation may be seen to apply to most other knots. Since such molecular dissymmetry may exist even in molecules all of whose carbon atoms are identical—indeed all of whose atoms are identical—the C-I-P convention cannot be made to apply.

**Figure 7:**
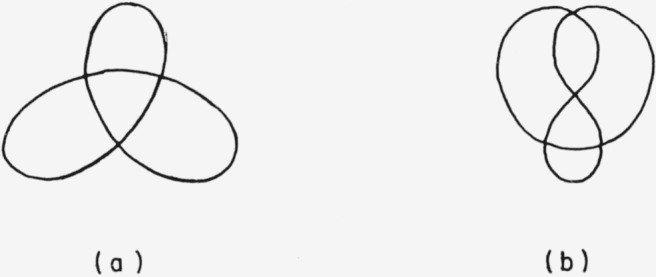
Normalized projections of (a) trefoil and (h) figure-eight knots.

**Figure 8:**
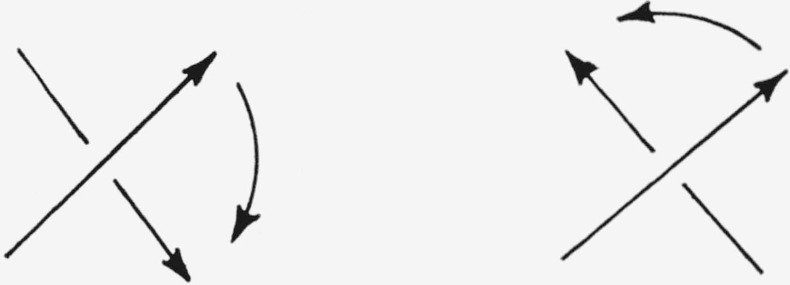
The two possible relative orientations of directed knot segments at a double point.

The designation of the absolute configuration of a knotted molecule must necessarily depend on the topology of the corresponding knot. A serviceable convention can be based on the normalized knot projections.^9^ For each knot there exists a planar projection 10 having a minimum number of intersections. Normalized projections of the trefoil knot and of the figure-eight knot are shown in figure 7. That which projects as an intersection in the planar projection shall be called a *double point* in the knot. At each double point one segment of the knot passes over another segment of the knot. If a direction is ascribed to the knot, then at each double point two situations are possible (cf. fig. 8). The projection of the upper segment must be rotated either clockwise or counterclockwise through an angle of less than 180° to bring it into coincidence with the projection of the lower segment. A *characteristic ϵ* shall be ascribed to each double point; *ϵ* = + 1 if the projection of the upper segment must be rotated clockwise to bring it into coincidence with the projection of the lower line segment, and *ϵ* = −1 if it must be rotated counterclockwise. Examination of figure 8 reveals that the characteristic of a double point is invariant: If the knot is turned over (viewed from the other side) or if the direction ascribed to the knot is reversed, then the value of the characteristic is still *unchanged*.

It is now possible to form the sum Ʃ*ϵ* over all double points of the knot. I propose that the absolute configuration of a knot be designated

(R)ifΣϵ>0

and

(S)ifΣϵ<0.

**Figure 9:**
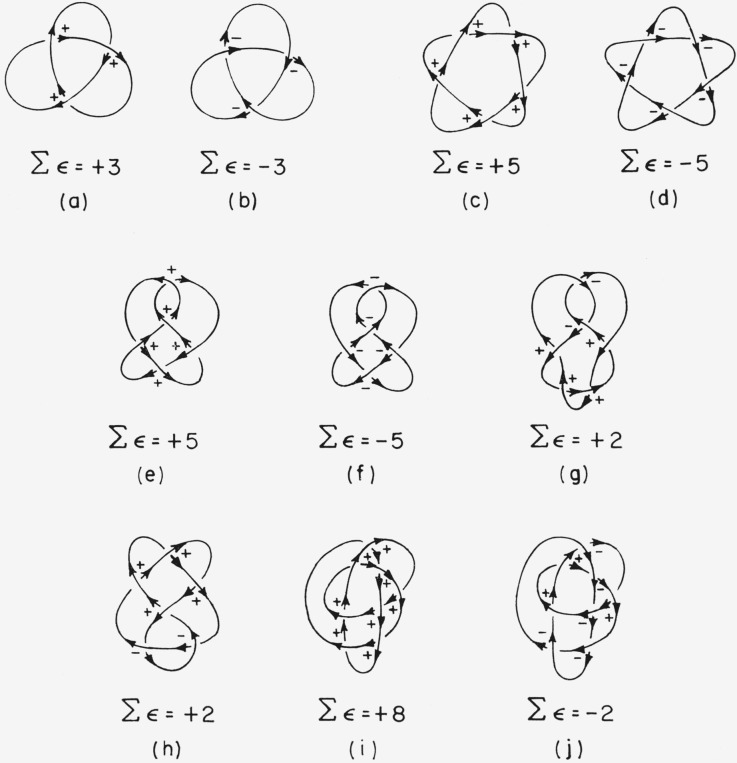
Examples of knots which differ from their mirror images. (A ± sign indicates each double point with a value of the characteristic *ϵ* = ±1. The total of the values of the characteristics is indicated under each knot. The arrows superimposed on the knots are for ease of determining the values of the characteristics and are *not* an attribute of the knots.)

By this convention the knots in figure 9 designated as (a), (c), (e), (g), (h), and (i) are in the (*R*)-form and knots (b), (d), (f), and (j) are in the (*S*)-form. Knots (a) and (b), (c) and (d), and (e) and (f) are enantiomeric pairs.

**Figure 10:**
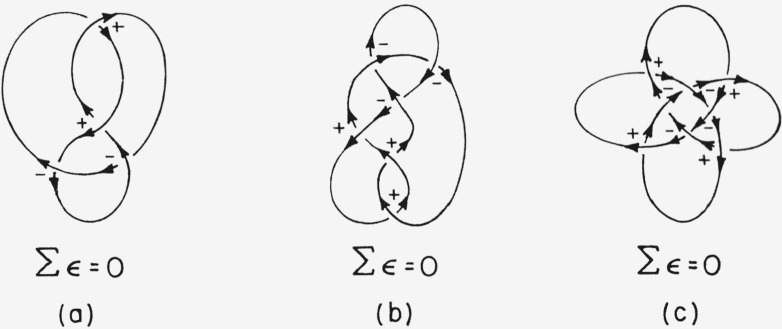
Examples of knots which are identical with their mirror images.

**Figure 11:**
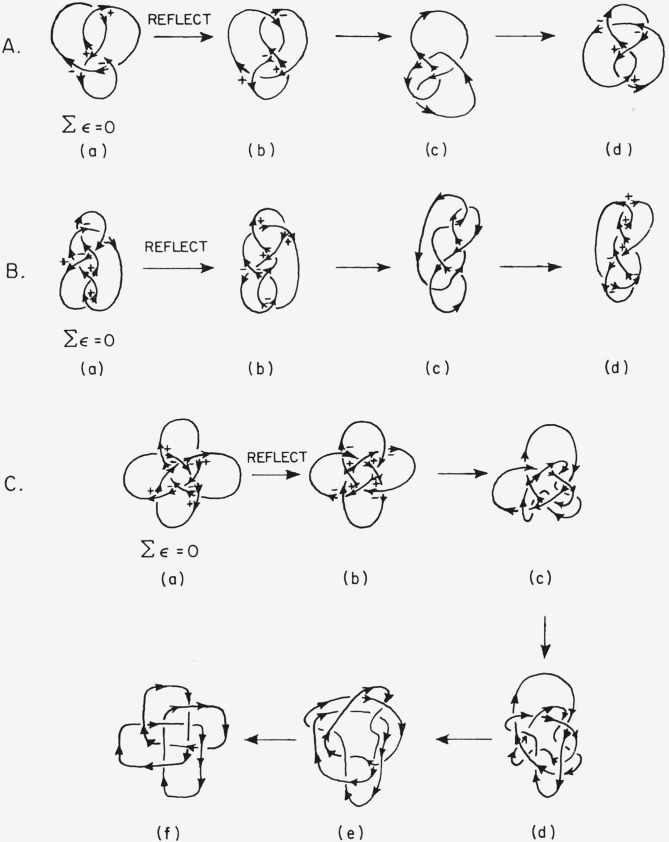
Successive distortions of the mirror images of knots (b) into the original knots (a).^*^ The knots of figures 11A and 11C are identical with their mirror images regardless of direction attributed to the knots. The knot of figure 11B is identical with its mirror image if and only if no direction is attributed to it. ^*^A: Rotate (d) 180° about a horizontal axis in the plane of the paper; B: rotate (d) 180° in the plane of the paper; C: rotate (f) 45° in the plane of the paper and smooth the curve.

For certain knots Ʃ*ϵ* =0 (cf. fig. 10). This is exactly as it should be, for precisely these knots are identical with their mirror images (cf. fig. 11). Note however that although knot (b) of figure 10 is not per se optically active, if the knot has an intrinsic direction then it exists as an enantiomeric pair (cf. fig. 11B). For this situation a further convention is necessary for assigning an absolute configuration. I propose the following *directed knot convention:*

1. Assign the positive direction to the knotted molecule such that one proceeds *to* the atom most senior by the sequence rule *from* its more senior neighbor.
2. Determine the largest possible number of successive double points along the knot in the positive direction which have *ϵ* = + 1. Define the molecule as
  (*R*) if an equal number of successive double points follow which have *ϵ* = −1, and
  (*S*) if a smaller number of successive double points follow which have *ϵ* = −1.

The two atoms under subrule (1) are to be members of the cyclic portion of the molecule when possible, otherwise they are to be the atoms nearest the cyclic portion which can define the direction. Thus the lactone V has its positive direction defined by proceeding from the carbonyl carbon atom to the oxy oxygen atom, and consequently, under subrule (2), it is the (*S*) isomer because after double points A, B, C, and D (with *ϵ* = + l) only double points E and F follow with *ϵ* = −1 before double point B is again reached. The positive direction of the chloroolefin VI is defined by proceeding from the terminal hydrogen atom to the chlorine atom, and the molecule shown is the (*R*) isomer since double points A, B, C, and A are followed by D, E, F, and D.

**Figure 12:**
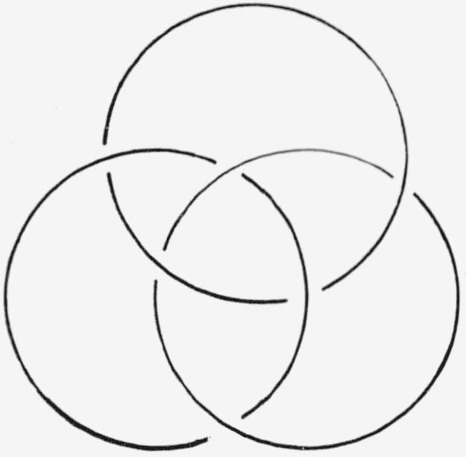
Borromean rings.

**Figure 13:**
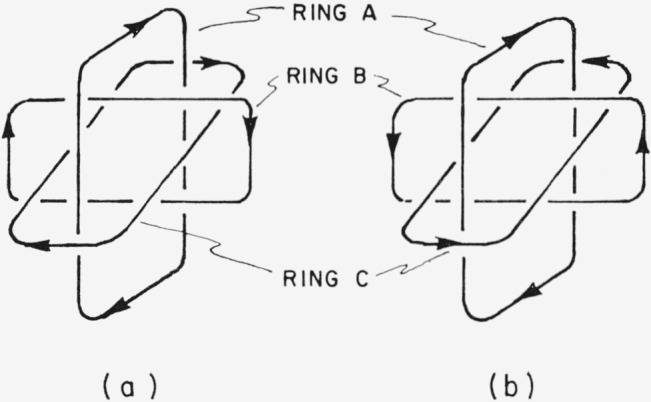
Borromean rings, showing the identity of mirror images. Reflection of (a) in a plane parallel to ring A yields (b), which when rotated 180° about the axis of ring A again yields (a).

It is worth noting that a set of Borromean rings is identical with its mirror image even if each ring has a direction attributed to it and each ring is different from the others, e.g., in the trilactone VII, 2,2′, 2″ trike to-1,1′, 1″-trioxa [67.63.59] ballantane. The identity of mirror images is more readily apparent when a set of Borromean rings is represented not as a “well-known brewer’s symbol” 11 (fig. 12) but in the alternative representation of figure 13.

## Molecularly Dissymmetric Diastereomers

The designation of the absolute configuration of a compound is fixed entirely by the stereochemical structure of the compound. However, for purposes of *citing* the absolute configuration it is generally necessary to couple the symbol (*R*) or (*S*) with the name of the compound and, in the case of compounds having several asymmetric centers, with the designation of the center of asymmetry. Thus we use such names as (2*R*:3*S*)-3-amino-2-butanol or (2*S*:3*R*)-3-hydroxy-2-butylamine. In the case of diastereomers involving molecular dissymmetry and a center of asymmetry, as in (*R*:5*S*)-5-methyl-2,3-heptadiene, VIII, it is readily apparent from the absence of a positional designation which symbol refers to the sense of the molecular dissymmetry. Nor can confusion exist in naming compounds having two similar axes or planes of dissymmetry, which would take the prefix (*R*:*R*)-, or *meso*-(or (*R:S*)-) to their names.

Possible confusion occurs when the absolute configuration of a molecule having dissimilar axes or planes of dissymmetry is cited. For names of such compounds I urge the following practice:

1. For compounds named fragment by fragment cite the sense of dissymmetry together with the fragment ; e.g., (*R*) -4-methyl-4-((*S*)-4-ketocyclohexyl) cyclohexanone dioxime and (*R*)-4-(2-(*R*:2*S*)-3,4-hexadienyl) cyclohexanone oxime.
2. For compounds named as a unit cite the senses of dissymmetry in the same order as that in which the parts of the molecule are cited; thus (*S*:*R*)-2,3,5,6-nonatetraene, but (*R*:*S*)-*α*-ethyl-*ω*-methyldiallenyl, for IX. An alternative procedure is to cite the atoms defining the axes or planes of dissymmetry, e.g. (2,4*S*:5,7*R*)-2,3,5,6-nonatetraene, but in the naming of very complex structures this can become excessively cumbersome.

## Footnotes

1 R. S. Cahn, C. K. Ingold, and V. Prelog, Experientia **12**, 81 (1956).

2 A. P. Terentiev and V. M. Potapov, Tetrahedron **1**, 119 (1957).

3 Cf., e.g.

- G. M. Dyson, A new notation and enumeration system for organic compounds, 2d ed., Longmans, Green and Co., Ltd., London (1949)—most recent edition published by Interational Union of Pure and Applied Chemistry Commission on Codification, Ciphering and Punched Card Techniques as Rules for I.U.P.A.C. notation for organic compounds, Longmans, Green and Co., Ltd., London (1961);
- M. Gordon, C. E. Kendall, and W. H. T. Davison, Chemical ciphering—a universal code as an aid to chemical systematics, Royal Institute of Chemistry of Great Britain and Ireland, London (1948);
- W. J. Wiswesser, A chemical line-formula notation, Thomas Y. Crowell Co., New York (1954); and
- H. W. Hayward, A new sequential enumeration and line formula notation system for organic compounds, Patent Office Research and Development Reports, No. 21, Patent Office, U.S. Department of Commerce, Washington (1961).

4 Contrary to the claims by partisans of one of the notation systems.

5 E. Wasserman, J. Am. Chem. Soc. **82**, 4433 (1960).

6 W. J. Ambs, Mendel Bull. **17**, Spring, 26 (1953).

7 H. L. Frisch and E. Wasserman, J. Am. Chem. Soc. **83**, 3789 (1961)

8 Cf. A.M. Patterson, L. T. Capell, and D. F. Walker, The ring index, 2d ed., American Chemical Society, Washington (1960), RRI 1967.

9 K. Reidemeister, Knotentheorie, Julius Springer, Berlin (1932); republ. Chelsea Publishing Co., New York (1948), p. 18 if.

10 Actually a set of equivalent projections.

11 E. Wasserman, Sci. Am. **207**, No. 5, 94 (Nov. 1962).

